# Meso-scale defect evaluation of selective laser melting using spatially resolved acoustic spectroscopy

**DOI:** 10.1098/rspa.2017.0194

**Published:** 2017-09-13

**Authors:** M. Hirsch, S. Catchpole-Smith, R. Patel, P. Marrow, Wenqi Li, C. Tuck, S. D. Sharples, A. T. Clare

**Affiliations:** 1 Optics and Photonics Group, University of Nottingham, Nottingham, NG7 2RD, UK; 2 Advanced Component Engineering Laboratory, University of Nottingham, Nottingham, NG7 2RD, UK; 3 Additive Manufacturing and 3D Printing Research Group, Faculty of Engineering, University of Nottingham, Nottingham, NG7 2RD, UK

**Keywords:** selective laser melting, microstructural texture evaluation, nickel superalloys, integrity, non-destructive evaluation

## Abstract

Developments in additive manufacturing technology are serving to expand the potential applications. Critical developments are required in the supporting areas of measurement and in process inspection to achieve this. CM247LC is a nickel superalloy that is of interest for use in aerospace and civil power plants. However, it is difficult to process via selective laser melting (SLM) as it suffers from cracking during rapid cooling and solidification. This limits the viability of CM247LC parts created using SLM. To quantify part integrity, spatially resolved acoustic spectroscopy (SRAS) has been identified as a viable non-destructive evaluation technique. In this study, a combination of optical microscopy and SRAS was used to identify and classify the surface defects present in SLM-produced parts. By analysing the datasets and scan trajectories, it is possible to correlate morphological information with process parameters. Image processing was used to quantify porosity and cracking for bulk density measurement. Analysis of surface acoustic wave data showed that an error in manufacture in the form of an overscan occurred. Comparing areas affected by overscan with a bulk material, a change in defect density from 1.17% in the bulk material to 5.32% in the overscan regions was observed, highlighting the need to reduce overscan areas in manufacture.

## 1. Introduction

Owing to the increasing interest in using additive manufacturing (AM) in high-value industries (i.e. aerospace, medical, tooling), there is a drive to employ novel materials in AM processes that yield parts with tailored properties [1,2]. This motivation arises from the shift in focus to AM processes being employed for customizable part production [3].

AM technologies consist of many processes that consolidate feed materials into three-dimensional structures using materials such as polymers, metals or ceramics [4]. Selective laser melting (SLM), described by a multitude of naming conventions [5], is currently being developed for new part manufacture for use in safety critical applications [6], but part integrity cannot yet be easily evaluated. SLM, the focus of this study, operates inside a closely controlled inert build chamber and enables the manufacture of metal parts from powdered feedstocks such as steels, titanium, aluminium and nickel alloys [7]. However, because structures are created through localized melting, defects can form. Structural defects, reported on in general SLM manufacture, include pores, both in the bulk of the material (spherical) due to trapped gasses [8] and in between layers (acicular) due to lack of material fusion [9]; unfused powder due to unstable power delivery or increasing oxygen content [10]; and metal balling due to high oxygen content in the build chamber [11]. In addition, when using hard-to-weld materials cracking can occur as a result of thermal gradients and the resultant stress concentrations [12]. Methods have been explored to reduce this issue by adapting the scan strategy and re-melting layers to normalize residual stresses [13,14]. Furthermore, post-processing heat treatments have been reported [15] that serve to normalize and re-form the microstructure. However, critical improvements are required with respect to process monitoring to help minimize the formation of these.

The nickel-based superalloy CM247LC (developed from MAR-M-247) [16] is of particular interest for aerospace engine manufacturers. This alloy was developed with lower carbon, titanium and zirconium content to reduce grain boundary cracking and increase medium temperature ductility. However, it exhibits poor weldability due to a high content of the $\gamma^{\prime }$-phase forming elements aluminium and titanium. Research conducted on this superalloy for use in SLM has attempted to reduce cracking by implementing ‘island’ scanning techniques [17]. Electron backscatter diffraction (EBSD) analysis showed that the majority of the cracks arose from the fine-grained boundary regions between the ‘islands’. Furthermore, it was observed that the laser scanning direction had a significant impact on the grain structure and crystallographic orientation of the samples [17]. In the work by Catchpole-Smith *et al*. [18], it was shown that, through optimization of the melting scan vectors, cracking of SLM-manufactured CM247LC parts can be reduced with a higher degree of process uniformity; crack origins and paths were repeatable under a wide range of process parameters.

Everton *et al*. [5] have recently reviewed the current state of inspection techniques for *in situ* process control. However, simply obtaining the location and type of a defect is often insufficient to determine the physical properties of performance parts. Destructive interrogation methods to measure microstructure are regularly used for parts created using traditional manufacturing techniques (e.g. forging or casting), and AM parts will be required to undergo the same interrogation for use in service. Methods such as EBSD and X-ray micro-computed tomography have been used to evaluate AM-produced parts destructively in order to gain statistical information about process parameters and feed materials [19]. It has been shown that microstructure information gained through EBSD can differentiate between skin scan and bulk scan areas [20], and that, under certain process parameters, grains form with a high aspect ratio in the *Z*-axis, yielding very fine grains in the *X*–*Y* section [21].

An emerging inspection technique known as spatially resolved acoustic spectroscopy (SRAS) has been used to determine the material microstructure and texture of industrially relevant materials [22,23]. It has been shown that SRAS measurements are in good agreement with EBSD measurements [24]. In a recent work [25], this non-contact laser ultrasonic inspection technique has been used to extract surface and subsurface pore information from specimens manufactured by SLM (Ti–6Al–4 V). Since the grain sizes of AM parts are expected to be much smaller than the effective resolution of SRAS, the acoustic scans reveal microstructural texture information. However, in current research, samples for SRAS have to be prepared by polishing the surfaces due to the method of data acquisition through a high-frequency photodiode. Work has been done on enabling the system to measure on as-manufactured surfaces by means of a photodiode array [26].

In this study, SRAS is used to analyse material defects on samples produced by SLM using the nickel-based superalloy CM247LC. In addition to providing surface defect information, SRAS is used to provide information on the variation in material texture. These material inconsistencies, not observable with optical methods, were identified and linked to surface defect density. The aim of this paper is to highlight the capabilities of SRAS as an evaluation method for investigating AM-produced parts using the nickel superalloy CM247LC.

## 2. Methodology

**Figure 1.**
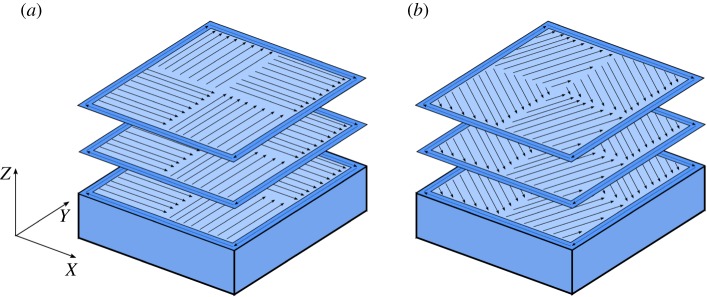
Scan strategies employed in the production of test samples: (*a*) an island scan with 0° hatch scan rotation and (*b*) an island scan with 30° hatch scan rotation. The arrows indicate the scan vectors and the darker outline indicates a skin scan. (Online version in colour.)

For this study, sample cubes were manufactured using a commercially available apparatus, a Realizer SLM50. SRAS and comparative analysis with optical microscopy (OM) were then undertaken. The samples were manufactured from CM247LC powdered feedstock (size distribution 15–100 µm; D10 = 20.0 µm; D50 = 36.4 µm; D90 = 57.1 µm), which is widely reported to be prone to defects within build. The chemical composition of CM247LC by weight per cent is given in table 1 [27]. The SLM manufacturing parameters were as follows: hatch spacing of 50 µm, laser spot size of 50 µm, laser power of 100 W, a scanning speed of 400 mm s^−1^ and a layer thickness of 25 µm. Each layer was produced using a hatching pattern sectioned into four equal ‘islands’ with a side length of 5 mm. The internal scan pattern within each island was angled at 0°, 15°, 30° and 45° to the island border, as shown in figure 1*a* (showing a 0° hatch scan) and figure 1*b* (showing a 30° hatch scan). Note that the ‘island’ borders in the scan strategy are at 90° to each other for all samples.

**Table 1.** Chemical composition by weight for the CM247LC nickel-based superalloy [27].

elements	Ni	Cr	Ti	Al	Mo	Co	C	W	Hf	B	Zr
CM247LC	Bal	8.10	0.70	5.60	0.50	9.20	0.05	8.50	1.40	0.015	0.015

After manufacture, the samples were sectioned in the *X*–*Y* plane as this enables a comparison between analysis processes, as well as a comparison of material fusion with regards to hatch scan angles. The sections were resin mounted, ground and polished to *R*_a_ ∼ 200 nm to reveal surface defects that were measured using OM and SRAS.

The SRAS instrument used for this study is described in detail elsewhere [22,23], and has previously been used to interrogate SLM samples [25,28]. The laser ultrasonic technique operates by generating surface acoustic waves (SAWs) with an incident pulsed grating pattern (i.e. generation patch). The grating period determines the SAW wavelength at the point where the wave is generated. The wave is quantified with a split photodiode detector measuring a detection laser which is incident on the sample at a point along the wave propagation path (approx. 8 µm from the generation patch). The SAW velocity is calculated using the known grating pattern wavelength and measured frequency. As the SAW velocity varies with the magnitude and directionality of the elastic properties of the material, it is possible to infer grain boundaries and crystallographic orientation. If the interrogation area (i.e. half the generation patch size) is much smaller than the expected grain size, then SRAS can be used to image individual grains and their boundaries. If the interrogation area is larger, or of the order of the size of the expected grains, then SRAS will produce microstructural texture information. Furthermore, it is possible to quantify the intensity of the incident detection laser, which can then be used to produce an optical image akin to a laser microscope. In its current operation, the SRAS system produces two distinct datasets: the optical image and a velocity map. In this study, the grating pattern wavelength and generation patch size used were 24 µm and 200 µm, respectively—micrographs were created using a raster scan of samples with a step size of 2 µm. The effective resolution of this inspection technique is based on the step size.

**Figure 2.**
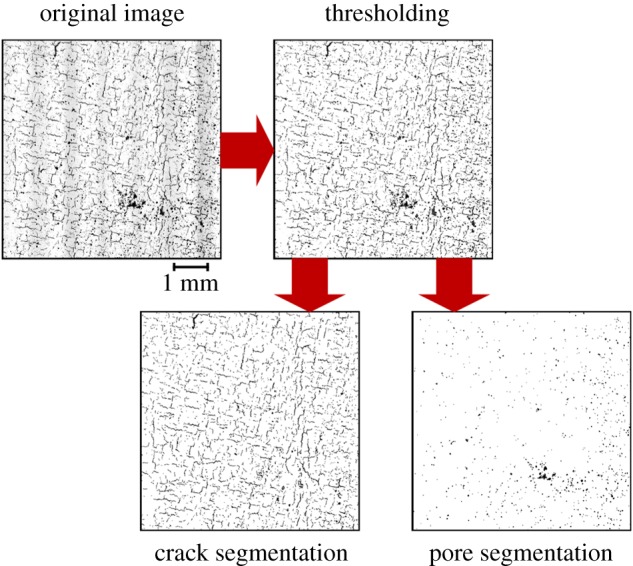
Micrograph analysis steps outlining the order of operations for segmentation of pores and cracks from the optical data of both measurement processes. (Online version in colour.)

Optical micrographs were taken using a Nikon Eclipse LV100ND microscope using a ×20 lens and the in-built image stitching tool was used to create compound images. Upon completion of data acquisition of the *X*–*Y* sections, the data were post processed using Fiji [29]. Data from both the optical microscope and the SRAS optical images were thresholded using the *maximum entropy thresholding* function with a range of 4–255 on an 8-bit bitmap image [30]. Steps were taken to segment the optical micrographs of both the SRAS and OM scans into pore-only defects and crack-only defects, as shown in figure 2. The separation threshold of cracks and pores was defined by an aspect ratio of 0.2 within the *f*_aspect_ function in Fiji. Using the SRAS optical images, it was possible to segment pores and cracks using different thresholding ranges. For each sample, four sectors (2000 × 2000 µm) were analysed to enable averaging of defect data.

## 3. Results

**Figure 3.**
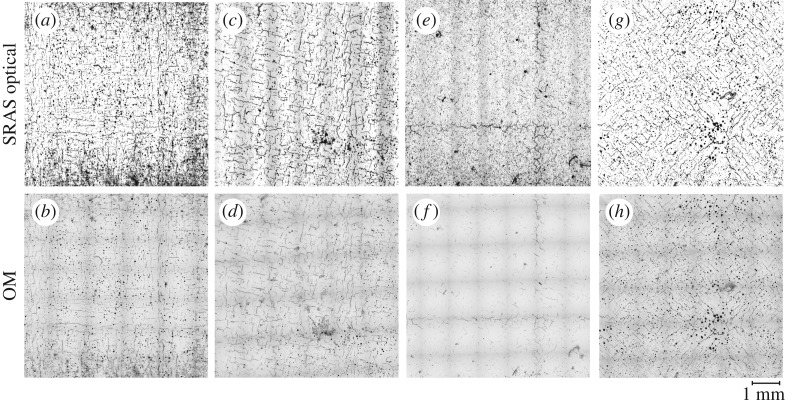
SRAS optical micrographs of (*a*) 0° sample; (*c*) 15° sample; (*e*) 30° sample; and (*g*) 45° sample and OM micrographs of (*b*) 0° sample; (*d*) 15° sample; (*f*) 30° sample; and (*h*) 45° sample. On SRAS, a distinct difference in signal intensity can be seen for different types of defects (pores and cracks) on the sample surfaces. The OM micrographs exhibit stitching artefacts over all samples.

Four samples at varying build parameters were produced as detailed in the Methodology. Analysing the layer integrity is useful for assessing the impact of AM process parameters; in this case, the hatching pattern and its rotation. Figure 3 shows both SRAS optical micrographs and accompanying OM micrographs of the prepared samples at section *X*–*Y*, where the SLM scan hatching pattern has been rotated by 0°(*a*, *b*), 15°(*c*, *d*), 30°(*e*, *f*) and 45°(*g*, *h*). The SRAS micrographs represent the signal intensity of the detection laser—both surface cracks and surface pores are present on all prepared surfaces. Through shape analysis and thresholding, it is possible to separate the location of each crack and pore.

**Figure 4.**
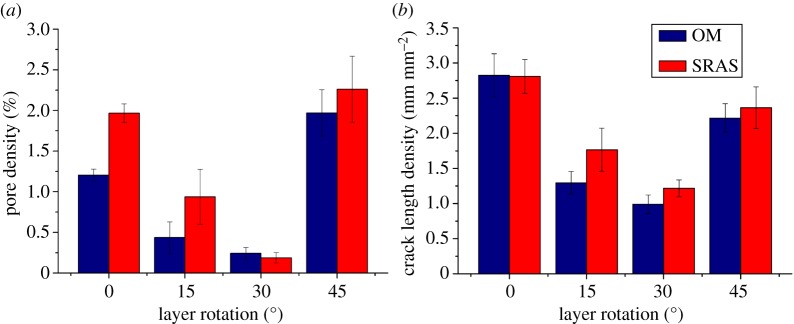
Defect quantification of the 0°, 15°, 30° and 45° samples calculated from OM micrographs and SRAS optical micrographs. (*a*) Pore density; (*b*) crack density. (Online version in colour.)

The optical datasets produced comparable defect density figures so a comparison of the effect of rotation of the melting laser hatching regime can be conducted. Quantified results of micrographs from both analysis techniques were obtained for comparison; the data presented in figure 4 show the densities of pores (*a*) and crack length density (*b*) identified using OM and SRAS on each sample. Data were obtained from four identical sectors on each sample.

It can be seen from the graphs presented in figure 4 that quantification of both pores and cracks, on average, is similar for both measurement techniques; the maximum error in defect densities between these is less than 0.75%. There are two reasons for the differences in the measurements. In SRAS analysis, a decline in signal intensity occurs when interacting with the outer edge of a defect that significantly extends down into the sample perpendicular to the scanned plane. The optical return of the detection laser is reflected at a shallow angle, reducing the proportion of the generated signal returning to the detector photodiode. For OM, a source of error comes from the image acquisition, where the shallow depth of field (numerical aperture of 0.45) prevents accurate focusing over large areas that are not perfectly planar when using image stitching within OM. Owing to these errors, any subsequent analysis operations, such as thresholding, will then falsify quantification data due to inaccurately defined edges of morphological defects.

**Figure 5.**
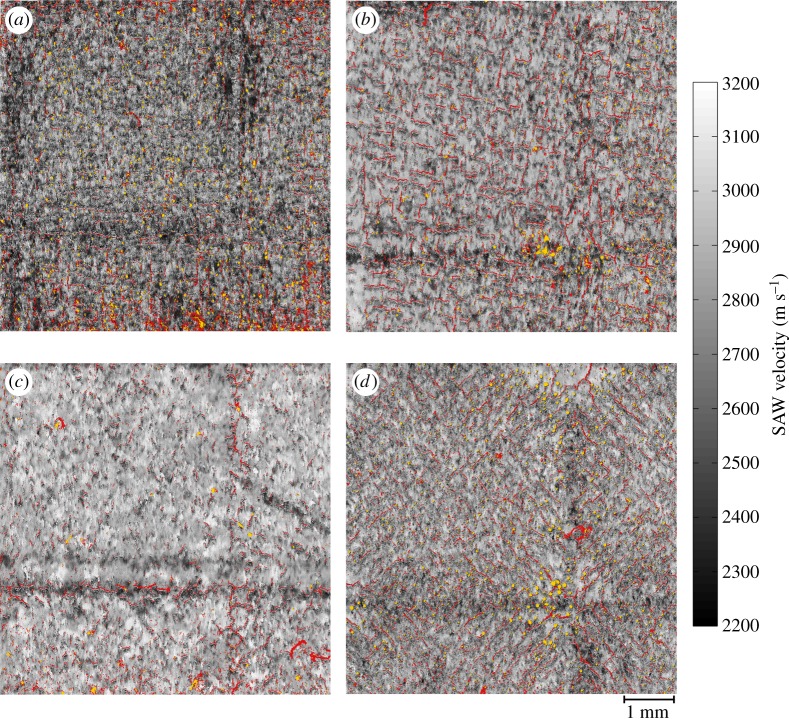
SRAS velocity maps of (*a*) 0° sample; (*b*) 15° sample; (*c*) 30° sample; and (*d*) 45° sample. Cracks and pores are highlighted as an overlay in yellow and red, respectively.

The main dataset provided by SRAS is the SAW velocity measured at each scan point; velocity maps of the samples are shown in figure 5*a–d* showing the 0°, 15°, 30° and 45° samples, respectively. The optical surface information has been overlaid to highlight cracks (red) and pores (yellow) that are present on the surface.

Since the expected grain size for each of the samples is of the same order of magnitude as the resolution of the SRAS instrument, it is not possible to identify individual grains or their boundaries. The data at each scan point represent the predominant velocity observed across the interrogation area; the resultant velocity map gives an indication of the microstructural texture of the prepared samples. Differences in appearances can be seen in figure 5 due to a change in the predominant SAW velocity measured. This can arise due to differences in the angle of the melting strategies. Furthermore, due to changes in defect densities, the image representation may become skewed (here, 30° sample versus 0° sample).

Disregarding the presence of defects present in the optical images, it is not possible to see any separation between the scan islands (described in figure 1). However, there is a clear drop in the velocity at the island interfaces seen at *X* = 5 mm and *Y* = 2 mm. This velocity shift gives an indication of an abnormality in the consolidation processes (relative to the other scanned areas).

## 4. Discussion

Defects in the form of cracks have been observed to form based on the employed melting strategy. A source for this is that residual stresses develop during contraction of the solidifying alloy as it cools selectively. Tensile stresses, therefore, act within the track in the planes aligned with the scan vector and directly perpendicular to the scan vector [31]. A schematic of the hatching scan vectors is shown in figure 1. As seen in figures 3 and 5, with scan angles of 0° and 45°, there is a large mismatch in scan angle at the intersection between the ‘islands’ and where the ‘islands’ intersect with the border. This results in a concentration of residual stresses in these regions and hence a higher propensity for the development of cracks, which is shown in the data presented in figure 4. At a hatch scan angle of 30°, both the pore density and crack density are the lowest of the datasets, while at 0° and 45° crack and pore density are significantly larger.

**Figure 6.**
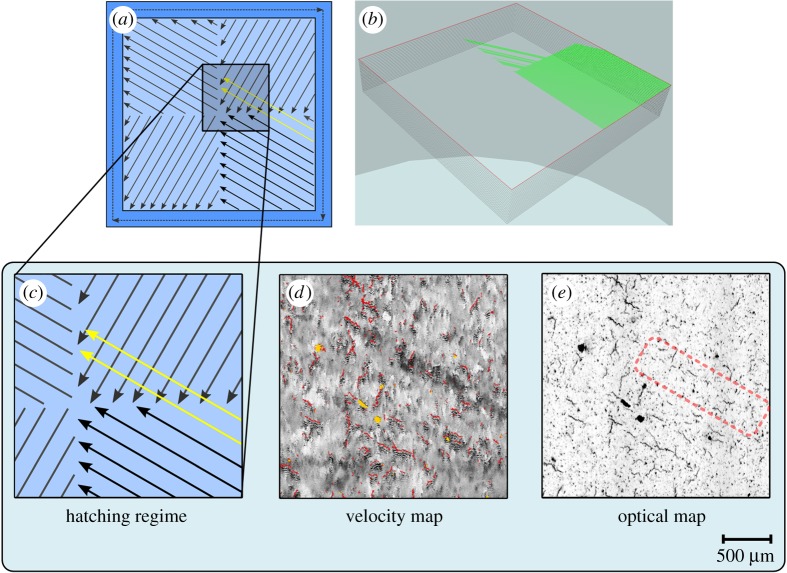
Melting laser scan error schematic showing: (*a*) the island scan pattern inconsistency observed in manufacture of the 30° sample; (*b*) an image from simulation of the build demonstrating the error in the build file; (*c*) a close-up of the overscan error of the melting laser; (*d*) the SRAS velocity map of that area; and (*e*) the corresponding SRAS optical map with the pattern inconsistency position highlighted. Note the lack of defect observed in the optical map.

During preparation for manufacture, the proprietary machine vendor slicing software induced a hatch fill error into the manufacturing code. As a result, the fill pattern had overshot the intended region; the melting scan in the south-east island had continued into the north-east island, as shown in the schematic in figure 6*a*. This error in manufacture was detectable when simulating the build process (figure 6*b*), but could not be identified by either OM or the optical return of SRAS (figure 6*e*). However, the SRAS velocity images show a drastic change in velocity in the affected sectors (relative to the surrounding area). This is most notable in the 30° sample as can be seen in figure 5*c* (5 mm < *X* < 7 mm, 3 mm < *Y* < 4 mm), with a magnified image shown in figure 6*d*. The ability to perceive where there is a change in the print process (e.g. overscan, underscan) relative to other sections in the build is useful for finding sources of defects. It allows the machine operator to investigate the cause of the process error to prevent the need for repairs or scrappage in future builds. Critically, *in situ* SRAS scanning of every layer would present the opportunity for *in situ* repair of components, mitigating the effect of the defects or removing them entirely.

**Figure 7.**
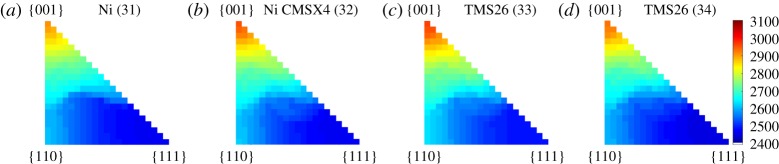
Average velocity maps of all plane families of nickel and three different nickel superalloys, which are calculated based on the elastic constants determined by (*a*) Salama & Alers [32], (*b*) Zhang *et al*. [33] and (*c*) and (*d*) Ichitsubo *et al.* [34,35], respectively.

In work by Carter *et al*. [17] it was shown that EBSD scans on CM247LC parts yielded comparable texture data to the findings presented here. It was observed that elongated grains with a strong {001} texture were observed in the central regions of the hatching islands, indicating a uniform melting strategy with grains growing in the *Z* direction. However, in island boundary zones a more chaotic and finer grain texture was observed with the crystallographic orientation deviating to {101} and {111}. In order to show that the findings presented here follow the same observations, the velocity response of texture changes for nickel alloys interrogated by SRAS has been simulated, with the method which was described by Li *et al*. [22]. This is shown in figure 7, where it can be seen that the average velocity gradually decreases as the orientation moves from {001} towards {101} and {111}; a single crystal nickel model and three nickel alloy models are presented which show a similar progression. Such lower SAW velocity at the island's interface indicates that the SRAS measurements agree with Carter *et al*.'s finding. Minute changes in texture orientation cannot be inferred due to the signal-to-noise ratio (±10%). However, based on the models, a cut-off velocity of approximately 2750 m s^−1^ can be defined to segment the crystallographic orientation of {001} from {110} and {111} as this is an equidistant point from all three primary plane families.

**Figure 8.**
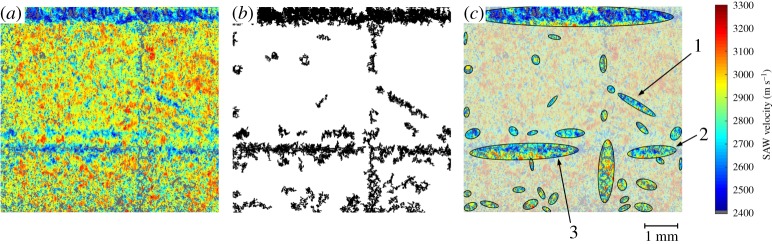
Sector analysis of a 30° sample. (*a*) The original scan data, (*b*) thresholding the velocity map below 2750 m s^−1^ and (*c*) extracting sector information through fitting ellipses. Note that the topmost fitted ellipse in (*c*) is an artefact that occurred during polishing that has been subsequently disregarded in the defect density analysis.

The SRAS velocity maps, presented in figure 5, show areas with a velocity of less than 2750 m s^−1^, segregating the island boundary areas and the areas that have been overscanned by the melting laser. This can be linked back to non-uniform heat input into the melting of the part, which can create anisotropic part quality in the samples. For manufacturing process stability, detection of this build quality-related non-ideal melting environment can prove helpful to enable early scrappage or avoid defect creation altogether. A scan of the 30° sample (figure 8*a*) has been processed to show how thresholding of the SRAS velocity data to reveal low-velocity sectors can be used for quantification of errors or defects in an AM environment. The algorithm processing steps consist of thresholding at a fixed velocity to reveal the low-velocity areas (figure 8*b*), followed by fitting ellipses to the defects to define the affected areas and perform a defect density comparison. This is shown in figure 8*c*, where best fitting ellipses have been superimposed on the original data to yield geometrical quantification of the non-ideal melting sectors identified.

Three exemplary zones have been defined in figure 8*c* to show that the non-ideal melting zones coincide with the hatching regime employed in the sample manufacture. This is most notable in area 1, which has an inclination of 30.8° to the *X*-axis, indicating a feature that occurred as a result of an overscan error (outlined in figure 6). Areas 2 and 3 have an inclination of 5.0° and 1.7°, respectively, indicating that this is an island boundary zone as it is closely aligned with the *X*-axis.

Through the determination of the non-ideal melting zones, a comparison in defect densities of the 30° sample can be conducted. A defect density of 5.32% was determined within the non-ideal melting zones, whereas the defect density in the central regions (bulk melting strategy) is 1.17%. This stark difference in defect density further supports the observation that the island boundary regions and overscan regions are not uniformly melted and should be controlled in part manufacture.

## 5. Conclusion

SRAS has been shown to be a viable analysis method for additively manufactured CM247LC parts. In comparison with OM, the optical signal return of SRAS has been shown to be in good agreement and can be used to differentiate between surface defects—pores and cracks. It was shown that the hatch scan orientation plays an important role in the defect densities observed, with a four-island 30° hatch scan resulting in the least amount of defects present on the surface—a defect density of 1.25%. In addition to the optical signal return, acoustic information can be obtained of the surfaces. Through a SAW velocity analysis it was shown that texture-based defects can be identified. This is highlighted on the 30° sample, where an overscan region was observed, arising through a manufacturing error as well as island boundary zones. This average change of texture has been linked with a change in crystallographic orientation and it was shown that quantification of these zones showed a change in defect density from 1.17% in the bulk material to 5.32% in the overscan regions.

For further implementation of this analysis technique, data obtained by SRAS can be used to inform the AM process about links between the identified problem areas and process parameters. To bring this measurement technique *in situ*, the rough surface measurement capability of SRAS needs to be optimized. This then will enable on-the-fly process changes and can allow automatic re-melting of these areas.

## Data accessibility

All data are presented within this paper.
